# Retraction Notice to: Stem cell and colorectal carcinogenesis

**DOI:** 10.25122/jml-2023-1009

**Published:** 2023-02

**Authors:** M Stoian, V Stoica, G Radulian

**Affiliations:** 1 Carol Davila University of Medicine and Pharmacy, Bucharest; Internal Medicine Department, Dr. Ion Cantacuzino Clinical Hospital, Bucharest, Romania; 2 Carol Davila University of Medicine and Pharmacy, Bucharest, Romania; Nicolae Paulescu National Institute of Diabetes, Nutrition and Metabolic Diseases, Bucharest, Romania

## Abstract

Article J Med Life 2016; 9(1): 6-11; PubMed ID: 27713769 has been retracted at authors’ request.

## RETRACTION

The authors hereby retract the article titled Stem cell and colorectal carcinogenesis [1] published in Journal of Medicine and Life, Volume IX, Issue 1, pages 6–11 in 2016 acknowledging significant errors in the literature review and data analysis presented in the article. We apologize to the readers, reviewers, and editors of Journal of Medicine and Life for any inconvenience or concern.

## References

[1] Stoian M, Stoica V, Radulian G (2016). Stem cells and colorectal carcinogenesis. J Med Life.

